# Automated de-identification of free-text medical records

**DOI:** 10.1186/1472-6947-8-32

**Published:** 2008-07-24

**Authors:** Ishna Neamatullah, Margaret M Douglass, Li-wei H Lehman, Andrew Reisner, Mauricio Villarroel, William J Long, Peter Szolovits, George B Moody, Roger G Mark, Gari D Clifford

**Affiliations:** 1 Laboratory for Computational Physiology, Massachusetts Institute of Technology, Cambridge, MA 02139, USA; 2 Harvard-MIT Division of Health Sciences & Technology, Cambridge, MA 02139, USA; 3 Computer Science and Artificial Intelligence Laboratory, Massachusetts Institute of Technology, Cambridge, MA 02139, USA

## Abstract

**Background:**

Text-based patient medical records are a vital resource in medical research. In order to preserve patient confidentiality, however, the U.S. Health Insurance Portability and Accountability Act (HIPAA) requires that protected health information (PHI) be removed from medical records before they can be disseminated. Manual de-identification of large medical record databases is prohibitively expensive, time-consuming and prone to error, necessitating automatic methods for large-scale, automated de-identification.

**Methods:**

We describe an automated Perl-based de-identification software package that is generally usable on most free-text medical records, e.g., nursing notes, discharge summaries, X-ray reports, etc. The software uses lexical look-up tables, regular expressions, and simple heuristics to locate both HIPAA PHI, and an extended PHI set that includes doctors' names and years of dates. To develop the de-identification approach, we assembled a gold standard corpus of re-identified nursing notes with real PHI replaced by realistic surrogate information. This corpus consists of 2,434 nursing notes containing 334,000 words and a total of 1,779 instances of PHI taken from 163 randomly selected patient records. This gold standard corpus was used to refine the algorithm and measure its sensitivity. To test the algorithm on data not used in its development, we constructed a second test corpus of 1,836 nursing notes containing 296,400 words. The algorithm's false negative rate was evaluated using this test corpus.

**Results:**

Performance evaluation of the de-identification software on the development corpus yielded an overall recall of 0.967, precision value of 0.749, and fallout value of approximately 0.002. On the test corpus, a total of 90 instances of false negatives were found, or 27 per 100,000 word count, with an estimated recall of 0.943. Only one full date and one age over 89 were missed. No patient names were missed in either corpus.

**Conclusion:**

We have developed a pattern-matching de-identification system based on dictionary look-ups, regular expressions, and heuristics. Evaluation based on two different sets of nursing notes collected from a U.S. hospital suggests that, in terms of recall, the software out-performs a single human de-identifier (0.81) and performs at least as well as a consensus of two human de-identifiers (0.94). The system is currently tuned to de-identify PHI in nursing notes and discharge summaries but is sufficiently generalized and can be customized to handle text files of any format. Although the accuracy of the algorithm is high, it is probably insufficient to be used to publicly disseminate medical data. The open-source de-identification software and the gold standard re-identified corpus of medical records have therefore been made available to researchers via the PhysioNet website to encourage improvements in the algorithm.

## Background

### Introduction

A wide range of medical research – from epidemiology to the design of decision support systems – relies on medical records [1]. For both legal and ethical reasons, it is necessary to preserve patient confidentiality. In the United States the Health Insurance Portability and Accountability Act (HIPAA) [2] specifies 18 specific categories of information that must be removed from medical records to be used in research. These categories of protected health information (PHI) include names, geographic locations (more precise than a state), elements of dates except years, social security numbers, telephone and fax numbers and medical record numbers, among others. See Table 1 for a complete list of HIPAA-specified PHI categories.

**Table 1:** PHI types defined by HIPAA

PHI Type	Notes
Names	**Both full and partial, but not initials**
Locations	**All geographic subdivisions smaller than a state, including street address, city, county, precinct, zip code, and their equivalent geocodes**
Dates	**All elements of dates (except years) for dates directly related to an individual, including birth date, admission date, discharge date, date of death**
Ages > 89 years	**All elements of dates (including year) indicative of an age over 89 years. Such ages and elements may be aggregated into a single category of age 90 or older**
Telephone numbers	
Fax numbers	
Electronic mail addresses	
Social security numbers	
Medical record numbers	
Health plan beneficiary numbers	
Account numbers	
Certificate/license numbers	
Vehicle identifiers	**Includes vehicle serial numbers and license plate numbers**
Device identifiers and serial numbers	**Not restricted to medical devices**
Web Universal Resource Locators (URLs)	
Internet Protocol (IP) address numbers	
Biometric identifiers	**Includes finger and voice prints**
**Any other unique identifying number, code, or characteristic**	**E.g., full face photographic images of full faces, scars or tattoos (and any comparable images)**.

The process of de-identification generally involves scanning the corpus of medical records line by line to identify all occurrences of PHI. This identification of PHI could be conducted manually by clinicians or persons familiar with medical terms, by automated de-identification software, or by a combination of software and expert oversight. In prior studies, we have demonstrated that manual de-identification by medical professionals is prohibitively time-consuming, expensive [3], and unreliable [4]. (We found that resident clinicians could de-identify at a rate of about 18,000 words, or 90 incidents of PHI, per hour.) De-identification performance tends to be highly variable and error prone [4]. Large-scale accurate de-identification therefore requires automated software that is fine-tuned to the structure of the text, the content of the medical records, and the specific requirements of a particular project.

Fortunately, automated de-identification can be not only more reliable, but also more efficient and far less expensive than manual de-identification.

In this article a body of work is presented that addresses the de-identification of free text medical records in the MIMIC II database [1], a large annotated database of cardiovascular and related signals and accompanying clinical data from intensive care units (ICUs) in the United States. All free text elements in this database have been scrubbed for PHI. In order to further reduce the chance of identifying a particular individual, we have also attempted to remove any information that may aid in the identification of attending clinical staff. Furthermore, instances of year-only dates were also classified as PHI, since all data in the MIMIC II database requires date-shifting, and the altered date must be consistent with all date types in the text.

Previous work in the field of de-identification has focused largely on highly-structured type-specific records [5-7,11-20]. MIMIC II medical records, however, include free-text notes with a highly variable structure and method of reporting, which cannot be de-identified by methods dependent upon a consistent and known structure. We have developed an automated de-identification algorithm that is suitable for a wide range of medical free text. Our de-identification efforts in this study centered on developing a specific tool to scrub free-text nursing notes and discharge summaries, and standardizing the evaluation of de-identification software. The gold standard re-identified corpus of medical records is available on request from the PhysioNet website [8,9], together with the de-identification algorithm under an open source license.

### Health Insurance Portability and Accountability Act (HIPAA) guidelines

In the United States, guidelines for protecting the confidentiality of health care information have been established in the Health Insurance Portability and Accountability Act (HIPAA) [2] which came into effect in April 2003. Medical records are said to be de-identified when the risk is "very small" that the information can be used alone or in combination with other reasonably available information to re-identify individuals associated with the records. This risk can be estimated and documented statistically for all the medical records in question, or the safe harbor approach can be taken to show that every record is free of the 18 specific categories of protected health information (PHI) defined by HIPAA, as detailed in Table 1.

### Performance measures for de-identification algorithms

The performance of de-identification algorithms is generally expressed in terms of recall, precision and fall-out. **Recall **is the proportion of PHI identified by the software (true positives) out of all instances of PHI in the text (true positives plus false negatives). **Precision **is the proportion of true positives among all terms identified as PHI by the software (true positives plus false positives). **Fallout **is the proportion of non-PHI terms mistakenly reported as PHI (false positives) out of all non-PHI terms (true negatives plus false positives). Each word is counted as a separate term for this evaluation. The terms *recall*, *precision*, and *fallout *are synonymous with *sensitivity*, *positive predictivity*, and *false positive rate *as these terms are defined in the context of other detection problems.

### Related work

There are relatively few published reports concerning the de-identification of unstructured medical free text, and specific algorithms are not usually made publicly available. Gupta *et al*. [5] devised a de-identification engine for pathology reports that uses a complex combination of dictionaries and text-analysis algorithms. Their approach locates useful (non-PHI) phrases and replaces the rest of the text with de-identified tags. For the identification of relevant medical phrases, the algorithm uses the Unified Medical Language System (UMLS) meta-thesaurus [24], a National Institutes of Health (NIH)-sponsored collection of medical vocabularies, some of which are considered standard for particular applications.

Sweeney [6] developed the Scrub system, which employs templates and specialized knowledge of the context to replace PHI in medical records. The system attempts to identify PHI using "common-sense" templates and look-up tables of examplary PHI. Sweeney's system also uses probability tables for template matching, detectors for medical terms to reduce false positives, tools to identify words that sound like other words (to account for spelling variations), and detectors for recurring terms. Sweeney's Scrub system identified 99–100% of the PHI in its author's test set. The false positive rate and details of Sweeny's test corpus are not available.

Sweeney later developed the Datafly system [7], which uses user-specific profiles, including a list of preferred fields to be scrubbed, and what external information libraries are permitted. The Datafly system is licensed to Privacert, Inc. [11] and specifics are therefore not publicly available.

Ruch *et al*. [12] developed a technique that uses sophisticated natural language techniques to tag words with appropriate parts of speech and a specialized semantic category known as MEDTAG. The technique then uses contextual rules based on the tags assigned to the text, using up to five-word groups and some "long-distance" (non-local) rules implemented as finite state machines. The algorithm then attempts to identify PHI in a limited region around words marked as 'Identity Markers'. The technique was developed for post-operative reports, laboratory and test results, and discharge summaries, written primarily in French, though with some documents in German and English. The system found 98–99% of all personally-identifying information in their test corpus.

Taira *et al*. [13] created an algorithm to identify patient name references from clinician correspondence, discharge summaries, clinical notes, and operative/surgical reports from pediatric urology records. Their algorithm uses a lexicon with over 64,000 first and last names and a set of semantic constraints to assign probabilities of a given word being a name. After scanning each sentence and classifying it according to the type of logical relation it contains, the algorithm then extracts the potential name based on that logical relation. This technique was shown to have a recall of 99.2%, but it is limited only to patient names and is not applicable to other categories of PHI.

Thomas *et al*. [14] developed a method that uses a lexicon of 1.8 million proper names to identify potential names and a list of "Clinical and Common Usage" words from the UMLS. The *Ispell *spell-checker dictionary [25] was also employed to reduce false positives. If a word is on both lists, a few simple context rules are used to classify the word. This method was tested on pathology reports and identified 98.7% of all names in their test corpus.

Berman [15] developed a technique for removing most PHI from pathology reports by excluding all terms that do not appear in the UMLS. Berman's algorithm parses sentences into coded concepts from the UMLS and stop-words, which are high-frequency structural components of sentences, such as prepositions and common adjectives. All other words, including names and other personally identifiable information, are replaced by blocking symbols, so that the output is totally stripped of non-medical and extraneous information. As Berman points out, the limitation with the concept-match scrubber is that it blocks too much, so the output is full of asterisks (the blocking symbol) and the text is hard to read'. Since publishing the concept-match scrubber, Berman has published a new scrubber algorithm based upon doublet (word pair) matching [26]. Berman's new approach parses through a text, matching every possible doublet (word-pair) in the text against the list of a list of approved identifier-free doublets (about 200,000). The doublet scrubber preserves, in situ, those text doublets that match against one of the doublets in the "safe" list. Everything else in the text is blocked (with an asterisk). This produces an output that is much more readable than the concept-match output and which is also fully de-identified. Although a significant improvement, much useful text is still blocked.

A de-identification system similar to ours is the one developed by Beckwith [16] was tested on a pathology report corpus containing 3,499 PHI identifiers and was found to remove all identifying words in pathology reports with a sensitivity of 98.3%. The 19 HIPAA-specified identifiers that were missed by Beckwith's system were mainly consult accession numbers and misspelled names. Unfortunately, the system does not perform as well on nursing progress notes and discharge summaries.

Miller *et al*. [17] developed a de-identification system for scrubbing proper names in a free-text database of indexed surgical pathology reports at the Johns Hopkins Hospital. Proper names were identified from available lists of persons, places and institutions, or by their proximity to keywords, such as "Dr." or "hospital." The identified proper names were subsequently replaced by suitable tokens.

Sweeney [18] examined four de-identification algorithms: the Scrub system which locates PHI in letters and notes, the Datafly II system which generalizes and suppresses values in field-structured data sets, *Statistics Netherlands*' *μ*-Argus system, and the *k*-similar algorithm. The Scrub system comprises a system of parallel detectors, each detector recognizing a specific type of explicit identifier in a field-structured database. The Scrub system accurately located 98–100% of all explicit identifiers, but the removal of only explicit identifiers did not ensure anonymity. The Datafly II system de-identifies entity-specific data in field-structured databases. The final outputs of the Datafly II system are anonymous, yet medically useful. In the *μ*-Argus system the data provider assigns to each attribute the amount of protection necessary. The *μ*-Argus system does not ensure an anonymous database, but results in a lower frequency of removal of useful information than the Datafly II system. The *k*-similar algorithm divides the text into groups of words so that each group consists of *k *or more of the most similar tuples (a finite ordered list of words). The similarity of tuples is based on a minimal distance measure derived from anonymity and quality metrics. Sweeney concluded that the Datafly-II system can remove too many useful phrases, the Scrub and *μ*-Argus systems can fail to provide adequate protection, and that the *k*-similar system provides a good trade-off between these two systems, providing "sufficient" anonymization and "minimal" loss of useful medical information. (It should be noted, however, that there is no generally accepted definition of 'sufficient' and 'minimal' for this application.)

Sibanda *et al*. [19] developed a semantic category recognition approach for document understanding that analyzes the syntax of documents. More specifically, a statistical semantic category recognizer is trained with syntactic and lexical contextual clues and ontological information from the UMLS. The semantic category recognizer identifies eight semantic categories in medical discharge summaries, e.g., test results and findings. The results confirm that syntax is important in semantic category recognition, and Sibanda *et al*. reported PHI classification recall and precision measures of above 90% using their test corpus.

Sibanda also developed a software package for de-identifying medical discharge summaries involving statistical models that employs local lexical and syntactic context [20]. Each word in a sentence was considered in isolation, and a Support Vector Machine with a linear kernel, trained on human-annotated data, was used to determine if a given word was PHI. The de-identification software identified at least 92.8% of PHI and misclassified at most 1.1% of non-PHI in four test corpora.

A very recent development has been a competition run at the first Workshop on Challenges in Natural Language Processing for Clinical Data to de-identify discharge summary free-text data [27]. Excellent performance was achieved through combining heuristics and statistical methods by György *et al*. [28] and Wellner *et al*. [29], with recall and precision performance in the range of 96%–98% and 98%–99% respectively. Their algorithms require large labelled training and test sets, however. Furthermore, their systems were trained on relatively well-structured data, such as discharge summaries and it is unclear how their approaches would perform on nursing progress notes, which are significantly less structured and grammatical than the discharge summaries. In contrast, the system presented here is evaluated using nursing notes, which are likely to be more challenging to de-identify. Evaluation of our system using discharge summaries similar to those used in [27-29], as described in this article, will allow a more meaningful comparison between our approach and others, especially if a common corpus can be used to evaluate multiple algorithms.

### Overview of this article

The following sections of this article set forth the design of an automated de-identification program, methods of testing the software, and evaluation and discussion of its performance.

## Methods

### Overview of the de-Identification approach

The pattern-matching de-identification approach described in this article is generally applicable to any free-text medical records. The algorithm uses the Perl language to perform lexical matching with look-up tables, regular expressions, and simple heuristics that perform context checks to identify and remove PHI.

The current approach de-identifies names (of patients, clinicians, visitors, and proxies), locations (including hospital names, building names within hospitals, town/city names, street addresses, zip codes, and PO Box numbers), dates (partial/full dates and years), telephone/pager/fax numbers, patient and doctor identification numbers (including social security numbers, medical record numbers, unique patient numbers, unique doctor identification numbers), email addresses, URLs, and any mention of age information for patients over 89 years of age. Additionally, the algorithm implements filters to remove references to ethnicity, and common holidays (such as Thanksgiving, Christmas, Ramadan, Hanukkah, etc.) that can be used to infer the date of events or the cultural and ethnic background of the patients. It should be noted that ethnicities, common holidays, clinical provider identifiers (such as names or pager numbers) and year-only dates, are not defined as PHI by HIPAA.

Though the current version of the algorithm is tuned to patterns observed in nursing notes and discharge summaries in the MIMIC II database, the approach is general and may be customized to work on other free-text medical records. The system provides a modularized design and a configurable interface that allows users to enable/disable each PHI filter module. Dictionaries can be modified and replaced without changes to the software.

### Dictionaries

The algorithm uses four types of look-up dictionaries:

• Known PHI look-up tables for known names of patients and hospital staff. The MIMIC II database includes full patient names associated with each medical record, permitting the extraction of (correctly spelled) full or partial names of patients specific to each record by direct matching. The hospital staff name list includes the names of the clinicians from the hospital where the data is collected.

• Potential PHI look-up tables for generic female and male first names, last names, last name prefixes, hospital names, locations and states. Names and locations are classified as "ambiguous" if they are also found on a list of standard English words obtained from Atkinson's Spell Checking Oriented Word Lists [23] or on the list of UMLS terms [24].

• PHI indicator look-up tables contain keywords or phrases that often precede or follow PHI terms. These PHI indicators serve as context clues such as titles ("Mr.", "Dr.", etc.), name indicators (such as "mother", "son", "proxy", etc.), location indicators (such as "Hospital", "Town", "Street", etc.), and age indicators (such as "age", "patient is", etc.).

• Non-PHI look-up tables contain dictionaries of "common words" or UMLS terms that tend to be non-PHI. A list of common English words is taken from Atkinson's Spell Checking Oriented Word Lists.

Since the contents of the look-up tables are separated from the algorithm itself, changing and supplementing the look-up tables is simple. If the notes are from a new local area, for example, the contents of the look-up tables with the names of local places can be changed. See Appendix C (Additional file 1) for a listing of the dictionary files. For a detailed description on how these dictionary files are compiled, interested readers are referred to [10].

### Algorithm overview

The process of de-identification involves scanning the medical notes line-by-line, dividing them into individual words separated by whitespace. The process then identifies occurrences of PHI using dictionary-based look-ups and regular expressions. PHI instances that involve numeric patterns, such as street addresses, PO Box numbers, dates and telephone/fax numbers, are identified by regular expressions based on numeric patterns as well as appearances of contextual keywords, such as "road" for street address or "pager" for pager number. Appendix B (Additional file 1) provides several example regular expressions in the Perl syntax.

De-identification of non-numeric tokens, such as names and locations, involves both dictionary look-ups and context checks to locate potential PHI. First, the algorithm performs a lexical match on each word in the text with dictionaries of PHI look-up tables to locate known and potential PHI, which is then labelled with the associated dictionary type. Second, the algorithm performs pattern matching using regular expressions that look for patterns with various context keywords, known as name or location indicators, to find more named entities. Simple heuristics are applied to qualify or disqualify ambiguous terms as PHI. In the following section on PHI filter modules for names, examples of such heuristics are given.

**Figure 1:**
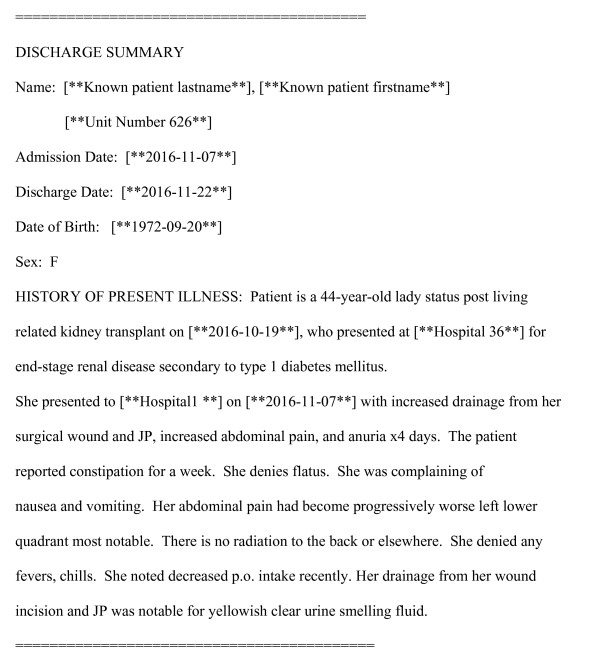
Example of discharge note after de-identification by the algorithm

The final step of the de-identification process involves replacing each PHI with a tag to indicate its corresponding category. Figure 1 illustrates an example of a de-identified discharge summary, in which PHI instances have been replaced by corresponding category tags. Note that all dates are shifted into the future by a patient-specific amount; see the section below on Dates.

### PHI filter modules

#### Names

Names directly identify patients and providers, and constitute the most risky PHI category. The algorithm uses both dictionary look-ups and context checks to locate potential names. The de-identification of names involves four basic mechanisms. First, the algorithm uses lexical matching with known PHI look-up tables to identify patient and clinician names. The MIMIC II database includes names of patient and clinicians. These names can therefore be extracted from each record by direct matching. The names in the medical notes could be spelled incorrectly, however, (e.g., "Willaim") or the patient may use a nickname (e.g., "Bill"), so the algorithm cannot rely solely on being provided with the name information. (Although an open source spell-checker was used in an early version of the algorithm [25], it was found that this gave little improvement in the sensitivity, and a large increase in the number of false positives.) Additionally, the identification and removal of the names of other people mentioned in the notes, including visiting relatives and the attending clinicians, is required. Thus, additional mechanisms are necessary to de-identify names.

The second mechanism identifies potential names within the text by lexical matching of words from the notes with all names in the lists of names obtained from the U.S. Census [22]. The names include nick names and abbreviated names such as Bill, Tom, Joe, Bob, etc. The potential names are classified as "ambiguous" and "unambiguous" names based on whether the names are also found on a list of standard English words obtained from the Spell Checking Oriented Word Lists [23] or on the list of medical terms from the UMLS. If a name is labelled "unambiguous", every occurrence of it will be removed from the text. If a name is "ambiguous", simple heuristics are applied to determine whether or not to remove it. These heuristics check for specific name patterns, such as a first name followed by a last name ("<first name> <last name>"), last name followed by a comma and then a first name ("<last name>, <first name>"), and full name with middle name or a middle initial (e.g., "<first name> <middle name> <last name>" or "<first name> <initial> <last name>"). For example, if an ambiguous first name is followed by an ambiguous last name in the text, then both words are removed as PHI.

Third, the algorithm performs a context check to identify additional PHI by examining words immediately preceding or following (1) names already detected in the text based on dictionary look-up, or (2) words that are PHI indicators (such as "Mr.", "Dr.", "name is", "daughter", "son", "husband", "wife", etc.). This mechanism allows the algorithm to identify additional names that are either misspelled or do not appear in any of the supplied name dictionaries.

The context check using names already detected in the text is based on the observed name patterns in the medical notes. For example, for each first name found in the text, the algorithm checks the immediately following word and applies heuristics to determine whether it should be removed; the following word is removed as a last name if it is not a recognized common word or UMLS term. Other identifying words, after which an ambiguous name is removed, include "Mr.", "Dr.", "wife", "friend", or "nurse".

Fourth, the de-identification software maintains a list of name instances found in the medical notes seen so far for a particular patient. After the algorithm has identified a list of names as potential PHI after processing a note, the entire note is re-scanned, and words that match the names in the PHI list found from this current note and also those identified from previous notes from the same patient are removed as PHI. This mechanism is motivated by the observation that the same names often reappear in the notes for a single patient. The patient's son may visit often, or the same clinicians may see the patient during her stay. While the first instance of a name is usually preceded by a name indicator, and thus can be de-identified by the third mechanism described above, subsequent mentioning of the same name may not be preceded by any apparent context keywords.

Names are currently replaced with a [**Name**] tag. If the software is able to determine the name types (first, last, or initial) based on dictionary look-ups and the name patterns (e.g., "last name, first name"), then the identified name PHI is replaced with name type tags accordingly, indicating whether the PHI is a full name, a female/male first name, a last name, and/or a name initial.

#### Dates

Medical discharge summaries and nursing notes tend to be rich in dates. The HIPAA regulations stipulate that all day/month combinations pertaining to patients, (e.g., birth, admission, discharge dates, etc.) be scrubbed during de-identification. Since it is difficult for automated software to determine whether a date pertains to a patient, all dates identified in the text are removed and replaced by a patient-specific offset. Dates generally follow specific formats, and the software attempts to match any of these formats in the text. Contextual information is also considered before identifying a portion of the text as date PHI.

Years are not considered PHI according to HIPAA regulations. However, years when taken in combination with other medical information may reveal when the patient experienced a landmark medical event. For example, the mention of "CABG 1996" in a nursing note divulges that the patient underwent a Coronary Artery Bypass Graft in 1996. This information may be used to substantially narrow down the set of patients to whom the medical record in question may pertain, thus increasing the risk of patient identification. All instances of years are replaced, in addition to the HIPAA-specified date formats, to maintain stringent de-identification standards.

The de-identification process replaces all PHI found with a PHI category tag. Dates, however, are necessary to track the patient's stay at the hospital and the evolution of his/her medical condition. For example, it is important for a medical researcher to know the interval in days between two events in the record, or the duration of the patient's stay in the hospital. The software therefore automatically re-identifies dates with a shifted date which preserves the day of the week and season to prevent inconsistencies with season-specific descriptions in the text and to prevent temporal errors or confusion due to relative temporal phrasing, e.g., "last winter" or "next Tuesday". Each date is shifted by a patient-specific random number of days that is consistent for the patient throughout all his or her de-identified medical files. The original date format in the text is preserved as much as possible in the re-identified text, e.g., month/year is replaced by a shifted month/year whereas an individual year is replaced by a shifted individual year. Dates of birth are treated in the same manner, preserving a patient's age.

#### Locations

HIPAA defines geographically precise location identifiers, which indicate a location smaller than a state, as PHI (see Table 1). Since the MIMIC II database contains patient information from local hospitals, neighboring locations are more likely than other geographic locations to appear as PHI. A list of neighboring locations was therefore compiled, and each word and phrase in the text is matched against this list to identify possible location PHI. Identified locations are replaced by [**Location**] tags.

#### Telephone, fax, social security, and other identification numbers

Patient and provider identities can be easily tracked down from telephone numbers, fax numbers, Social Security Numbers (SSN), medical identification numbers and medical record numbers. In fact, the level of risk associated with released SSNs can be as high as the risk associated with released full names.

However, patient-specific alpha-numeric identifiers can often resemble medical data. The de-identification software therefore checks the text for specific numerical formats, making sure to exclude medical data that may have similar formats, e.g., heart rates, blood pressures and blood gas data. For example, numeric patterns such as XXX-XXXX are generally identified as telephone numbers except when preceded by medical terms such as "SVR", "VT", "Tidal Volume" etc. The dictionary of these terms was compiled from repeated searches through the MIMIC II database for abbreviations.

#### Ages over 89

Hospital patient populations generally include few subjects over the age of 89 and therefore the HIPAA regulations require lumping of all ages over 89 into a single category. The de-identification software searches for either numerical or text patterns that fall within an age range of 90–125. For example, it will identify an age expressed either as '95', 'ninety-five' or 'ninety five'. The upper limit of the age range is introduced as a sanity check since it is highly unlikely that a patient's age will exceed 125. Additionally the software examines the textual context to determine if candidate text actually enumerates an age. Only numbers between 90 and 125 inclusively, that are either preceded or followed by words such as "age", "patient is", "years old", "yo", etc., are identified as PHI. The identified age is then replaced by a general [**Age over 89 **] tag that aggregates all ages over 89 into a single group to preserve confidentiality, but that still presents the age information to enable a medical understanding of the patient's condition.

#### Miscellaneous PHI categories

Not only must patient information be removed from the MIMIC II text files, but also information specific to the hospitals and its providers. Hospital-specific information can narrow down the subset of patients to whom a record may pertain. For example, the subset of 49-year-old patients in Ward A of Hospital X is significantly smaller than that of 49-year-old patients anywhere in a given state. To this end the following additional PHI categories have been added: provider names, hospital names, ward names, and any other HIPAA categories specific to hospitals and providers. The software de-identifies these categories using lists of known PHI, pattern-matching rules and context information.

The software searches for a wide range of miscellaneous PHI types that are not included in the gold standard corpus, which are listed in Appendix A (Additional file 1). In particular, subroutines exist which look for email and IP/URL addresses (using contextual clues such as "@", "http", "http", "://", "www.", "web.", ".org", ".com" and ".net").

## Data sets

The MIMIC II database is an annotated database of physiologic waveforms and related signals and accompanying clinical data from intensive care unit (ICU) patients. The database includes physiologic signals, free-text medical records, laboratory test reports, etc., for over 17,000 patients [1]. Approval for the collection and use of the data in this study was obtained from the appropriate institutional review board (IRB).

To evaluate the de-identification approach described in this article, a randomly selected subset of the nursing progress notes was extracted from the MIMIC II database. The nursing progress notes are unstructured free text typed into a clinical information system by the nurses at the end of each shift. The notes include observations about the patient's medical history, his/her current physical and psychological state, medications being administered, laboratory test results, and other information about the patient's course in the ICU.

Nursing notes appear to be significantly more challenging to de-identify than other forms of medical notes, such as discharge summaries and radiology reports. In nursing progress notes, the clinical staff frequently employs technical terminology, non-standard abbreviations, ungrammatical statements, misspellings, and incorrect punctuation and capitalization.

The de-identification approach described in this article was evaluated using two different corpora of nursing notes. The first corpus, consisting of 2,434 nursing notes, was fully de-identified and then re-identified with surrogate PHI by multiple experts to provide a gold standard for the de-identification software [3,4]. This gold standard corpus was used to fine-tune the software during its iterative development process. In order to test the software on data not used for development, a second set of 1,836 nursing notes, known as the test corpus, was prepared. PHI missed by the software was identified by human annotators to obtain an estimate of the recall performance of the approach detailed here. In the following sections, the gold standard corpus and the test corpus are described in more detail.

### Development of the gold standard corpus

A corpus of nursing notes was thoroughly de-identified manually and then re-identified with surrogate PHI (similar in nature to each PHI type removed) to create a gold standard for developing and testing de-identification software [3,4,10]. The names in the corpus were replaced with names adapted from publicly available lists of names with randomly swapped first and last names. Locations were replaced from randomly selected small towns in a different part of the country. For details of the re-identification process, interested readers are referred to Douglass *et al*. [4,10].

The gold standard corpus consists of nursing notes from the medical records of 163 patients selected randomly from the MIMIC II database [1]. All the nursing notes associated with these 163 patients were used, comprising 2,434 notes, with approximately 334,000 words. Of those notes, 99 were selected at random for manual "enrichment" to include text that is especially difficult to de-identify (such as "Wilson's disease" and "Parkinson's tremor") and to include more instances of PHI.

Three clinicians from local hospitals independently performed a manual review of the notes, labelling and classifying all PHI. A highly sensitive prototype de-identification algorithm was then used to locate any further PHI that the clinicians may have missed. A fourth clinician then reviewed all the results and adjudicated all disagreements. Each PHI was also re-identified by the same adjudicator using lists of suggested replacements drawn from type-specific dictionaries. The resulting gold standard corpus includes a list of all PHI in the original corpus, and is considered to have an almost perfect recall of 1.0 and precision of 1.0. Table 2 details the performance of a single clinician, the union of two clinicians, and the union of three clinicians de-identifying the corpus, with respect to the gold standard corpus. The results of de-identification varied from clinician to clinician, with recall ranging from 0.63 to 0.94. (Definitions of recall and precision are given in the Performance Measures section.)

**Table 2:** Clinician de-identification performance.

		Min	Max	Mean
1 person	Recall	0.63	0.94	0.81
	Precision	0.95	1.00	0.98
2 people	Recall	0.89	0.98	0.94
	Precision	0.95	0.99	0.97
3 people	Recall	0.98	0.99	0.98
	Precision	0.95	0.99	0.97

The gold standard corpus contains a total of 1,779 PHI elements. Of these, 211 were introduced manually into the enriched text. Before enrichment, there were about 120 words per note, 15 notes per patient and about 0.64 pieces of PHI per note.

Table 3 provides the frequency and distribution of each category of PHI in the original gold standard corpus (before enrichment), the number of enriching PHI added in each category, and the total PHI count and distribution in the resulting gold standard corpus (with enrichment). The PHI in the gold standard corpus fall into the following categories: name, date, location, phone number, age over 89, and other. The name category is further divided into the following sub-categories: patient names, patient name initials, relative/proxy names, and clinician names. The date category is divided into dates with day, month and/or year, and dates with reference to year only. The phone numbers include telephone numbers, cell phone numbers, and fax/pager/beeper numbers. The locations include hospital names and city/town names. Three instances of PHI found in the corpus were classified as "undefined", and are alpha-numeric patterns and numbers found in the notes that have un-identified type/meaning or are ambiguous in terms of whether they should be considered PHI. For example, one of them is a hospital policy number with an alphanumeric pattern that could potentially be used to infer the identity of the hospital.

**Table 3:** PHI category breakdown in gold standard corpus.

PHI Type	Original Count/Distribution	Added PHI (Enrichment)	Total Count/Distribution After Enrichment
Patient Name	34 (2.17%)	20	54 (3.04%)
Patient Name Initial	2 (0.13%)	0	2 (0.11%)
Relative/Proxy Name	125 (7.97%)	50	175 (9.84%)
Clinician Name	518 (33.04%)	75	593 (33.33%)
Date (not year)	475 (30.29%)	6	482 (27.09%)
Year	42 (2.68%)	4	46 (2.59%)
Location	328 (20.92%)	40	367 (20.63%)
Phone	37 (2.36%)	16	53 (2.98%)
Age over 89	4 (0.26%)	0	4 (0.22%)
Undefined	3 (0.19%)	0	3 (0.17%)

Total	1,568	211	1,779

Note that the 1,779 PHI instances in the gold standard corpus include both critical and non-critical PHI categories. (Critical PHI are personal health information as defined by HIPAA [2]. Non-critical PHI, such as clinician names, name initials, and years, not defined as PHI by HIPAA.) Of the 1,779 PHI, about 36% are non-critical PHI. While names in general account for more than 45% of the overall PHI, approximately two thirds of the names in the corpus are clinician names.

The gold standard corpus provided the basis for contextual evaluation and improvement of the de-identification software during its iterative development process. The re-identified gold standard corpus with its associated surrogate PHI information is available via PhysioNet [8,9] under a limited data use agreement. (The data use agreement requires the user to certify that they will not attempt to re-identify any of the individuals in the corpus, or distribute the data to anyone else.) Appendix A (Additional file 1) lists the PHI tags the software can generate.

### Test corpus

In order to test the algorithm on data not used in the development of the algorithm, a second set of nursing notes was selected. This "test" corpus consists of 1,836 nursing notes (and a total 296,400 words) randomly drawn from a subset of 123 patients in the MIMIC II database. In contrast to the gold standard corpus, the PHI in these notes is not re-identified and therefore the occurrences of PHI appear in their original form. Furthermore, no attempt was made to identify true positives in this test data, since the primary goal is to identify the PHI that escapes the de-identification algorithm. (A true positive rate can be as low as 80% or 90% without substantially affecting the readability of the notes [10].) The main concern here is to remove PHI robustly, with the understanding that the loss of some useful data is inevitable. An empirical assessment of the 'usefulness' of the data is application dependent and outside the scope of this article.

Therefore, instead of obtaining a full PHI count on the notes, human annotators were asked to identify only missed PHI, to allow us to estimate the false negative rate (which indicates the algorithm's recall). An early prototype of the de-identification software described here [4] was applied to the test data to generate a preliminary set of scrubbed nursing notes. Each of 14 reviewers was then assigned approximately 130 of these scrubbed nursing notes and was charged to identify any PHI remaining in the scrubbed text. (A financial incentive was offered to the reviewer who found the largest number of false negatives.) Reviewers labelled each of the missed PHI with the appropriate PHI category. The results of the findings are presented in the in the following results section.

### Evaluation criteria

The performance of the algorithm was analyzed on both the gold standard corpus and the test corpus. However, these analyses differed because the test corpus was annotated only for false negatives (i.e., missed PHI) and full human consensus annotations are only available for the gold standard corpus.

For the evaluation using the gold standard corpus, the algorithm was supplied with the re-identified first and last names of all 163 patients, a list of re-identified clinician names, local town names, and local hospital names, as well as a list of generic names (from Census data [22]), popular American names, and generic locations (e.g., major U.S. cities). Note that no name dictionary is usually available for names of visiting relatives or proxies. As a result, the algorithm relies entirely on pattern matching using a list of generic names to de-identify names of relatives and proxies.

Since false positives are not available in the test corpus, only the number of false negatives per PHI category is reported, and the PHI frequency distribution observed in the gold standard corpus is used to compute an estimated recall value for the test data.

## Results

### Performance on re-identified gold standard corpus

In this section, the algorithm's performance is examined on the public version of the gold standard corpus, which was used for the iterative development of the algorithm described in this article. The filtering performance for each different type of PHI is reported.

The de-identification output was evaluated using all 1,779 PHIs in the gold standard. The overall recall, precision, and fallout of the algorithm are 0.967, 0.749, and 0.002 respectively. The PHI types were also divided into 'critical' and 'non-critical' PHI. Critical PHI was defined as all PHI types listed by HIPAA, and 'non-critical' PHI as the remaining categories that were included to make malicious re-identification even more difficult. Excluding non-critical PHI from the evaluation does not change the algorithm's recall significantly; the recall of the algorithm on critical PHI in the gold standard corpus is 0.961.

Table 4 lists the number of PHIs, false negative count, recall, and precision of the de-identification algorithm in each category. The **per-category recall **for category *i *is defined as the number of true positives in category *i *identified by the software divided by the number of PHIs in that category. The **per-category precision **for category *i *is defined as the number of true positives in category *i *divided by all PHIs located by the algorithm using the filter specific to category *i*. No per-category precision is reported for PHI in the "Undefined" category, since these PHIs are extremely infrequent or absent from the gold standard corpus. Note that, in some cases, a word may be declared as PHI by multiple filter types in the algorithm. For example, the word "Calvert" in Calvert Hospital may be de-identified by both the name and location filters. Thus, for the per-category precision, the list of terms used to generate the denominator in the metric are not necessarily mutually exclusive across categories.

**Table 4:** Performance on gold standard corpus.

PHI Type	PHI sub-type	Count	# FNs	# FNs per 100,000 words	Per Category Recall	Per Category Precision
Name	Patient Name	54	0	0	1.00	
		
	Patient Name Initial	2	2	0.598	0.00	
		
	Relative/Proxy Name	175	4	1.195	0.977	
		
	Clinician Name	593	3	1.494	0.995	0.725

Date	Date (not year)	482	26	7.769	0.946	
		
	Year	46	11	3.287	0.761	0.713

Location		367	10	4.482	0.973	0.922

Phone		53	0	0	1.00	0.898

Age over 89		4	1	0.299	0.750	0.600

Undefined		3	2	0.598	0.333	N/A

Overall		1779	59	19.720	0.967	0.749

(FNs are false negatives and N/A indicates not applicable)

The algorithm is able to de-identify all references to the first and last names of the patients that appeared in the gold standard corpus. The algorithm has a recall at or above 0.95 in most of the critical PHI categories, including patient names, visiting relative names, dates (not year), locations, and phone numbers. The only exception is the 'age over 89' category, where only four PHI instances in that category are available for evaluation. The algorithm does not perform as well in name initials and years, which are not PHI according to HIPAA regulations, and are classed as non-critical PHI.

Although the precision of the software (0.75) is relatively low in comparison to manual de-identification (average precision 0.98 from one human de-identifier), it should be noted that the readability and information content of the de-identified notes are not significantly compromised. Of the false positives that were generated, about 38% are numeric patterns, most of which are physiological measurements that are available elsewhere in the MIMIC II database as either low- or high-resolution trend or waveform data. Many of these text based false positives are from random, misspelled words (e.g., "has" misspelled as "hass" will be de-identified as a name). Observations based on the de-identified medical notes generated from the software suggest that such misspelled words, when removed from the notes as false positives, usually have little impact on the readability of the notes, as they usually do not convey critical information about the patients.

Words that appear as false positives at high frequency are usually common words or medical terms that are also potential names, such as "MAE" (acronym for "moving all extremities") and "will". The top 5 most frequent non-numeric false positive terms (and their frequencies of occurrence in parenthesis) are: MAE (10), AND (6), WILL (5), LOS (4), and ADA (3). (LOS means "Length of Stay", ADA means "American Diabetes Association".) Medical terms and common words that appear in the UMLS and common word dictionaries are not removed as PHI unless they are potential names and are preceded or followed by name indicators (such as "daughter" or "friend") or another word that is also a potential name. For example, while "MAE" in general is left intact in the notes, "PERL, MAE" (pupils equal, reactive to light, moves all extremities) will be labelled as PHI (and replaced by a tag) since "PERL" is a potential name.

### Performance on test corpus

In this section, the performance of the software on the test corpus is detailed. The PHI elements in these notes were not re-identified and appeared in their original form. Instead, the false negative count was calculated, since minimizing the number of missed PHI is crucial in protecting patient confidentiality. The manual evaluators categorized the false negatives into their corresponding PHI categories. The results are summarized in Table 5.

**Table 5:** Categorization of algorithm false negatives by PHI type on test corpus.

PHI Type	# False negatives in 296,400 words/1,836 nursing notes	# False negatives per 100,000 words	Recall
Full name	4 †	1	
Last name	14 †	5	
First name	31 †	11	
Location (not street address)	7	2	
Full date	2	1	Unknown
Partial date	9	3	
Year	8	3	
Age over 89	3	1	
Overall	78	27	0.94 (estimated)

† None of these names were actually patient names, and therefore were *non-critical *PHI.

None of the full or last names that escaped the de-identification software were associated with patients. Furthermore, at the time of the evaluation, the software did not use the patient and provider names available in the MIMIC II database.

The software performs well at identifying HIPAA PHI types such as full names, full dates and ages over 89. Only one full date was missed, which although can present a potential problem, may not be critical if other associated dates are time-shifted. General locations (not street addresses), partial dates and years pose limited risk, and are recognized as PHI with reasonable effectiveness. Based on the frequency of PHI instances observed in the gold standard corpus (before PHI enrichment), an estimated 474 instances of PHI per 100,000 words should exist in the test corpus. This yields an estimated recall of 0.943 for the algorithm's performance on test data, where a total of 27 false negatives were observed per 100,000 words, or 0.55 false negatives per patient (see Table 5). However, over half these instances of missed PHI are part of the extended (non-HIPAA) PHI categories.

### Performance without customized dictionaries

One question that relates to the generality of the approach described in this article is how much the algorithm's power in de-identifying names and locations relies on dictionary-based look-up with known PHI, versus pattern matching using PHI indicators and look-ups using generic dictionaries. While using customized name and location dictionaries of known PHI types ensures the removal of those PHI occurrences from the medical text, this approach also makes the algorithm's performance dependent on the quality and completeness of the dictionaries supplied by the users.

In this section, the performance of the algorithm is tested by running the de-identification algorithm on the gold standard corpus without any customized dictionary of patient names, clinician names, local town names, and hospital names. The algorithm, however, is supplied with a dictionary of generic names and locations (e.g., major cities in the U.S.).

The results show that, as expected, the performance of the algorithm is lower without the customized dictionaries. The overall recall and precision of the algorithm drop to 0.834 and 0.725 respectively, although there is no significant change to the fallout value, which remained in the 0.002 range. The false negative count and recall for each PHI category are summarized in Table 6.

**Table 6:** Performance without customized dictionary on gold standard corpus.

PHI Type	PHI sub-type	Count	# FNs	Per Category Recall	Per Category Precision
Name	Patient Name	54	1	0.981	
	Patient Name Initial	2	2	0.00	
	Relative/Proxy Name	175	5	0.971	
	Clinician Name	593	24	0.973	0.731
Date	Date (not year)	482	26	0.946	
	Year	46	11	0.761	0.712
Location		367	231	0.371	0.840
Phone		53	0	1.00	0.898
Age over 89		4	1	0.750	0.600
Undefined		3	2	0.333	N/A
Overall		1779	295	0.834	0.725

(FNs are false negatives and N/A indicates not applicable.)

It should be noted that the de-identification algorithm performs very well in all names even without dictionaries of specific patient and clinician names; the recalls for all name categories remain above 0.95, suggesting that pattern matching and look-up on generic name dictionaries reap the majority of the performance benefits in these categories. This is due to the fact that there is a relatively consistent pattern to most of the names in the corpus. Patients' and doctors' names are mostly preceded by titles such as Mr., Ms., and Dr. Most of the relative/proxy names are preceded by keywords, such as "daughter", "son", "husband", or "proxy", etc., when they appear for the first time in the corpus. In addition to performing lexical matching with the dictionary (when one is provided), the algorithm uses the titles and keywords as well as a list of generic names for de-identification.

In contrast, the location detection subroutine performs significantly worse without the local town and hospital names dictionaries; the recall in the location category drops from 0.97 to 0.37. This is partly due to the fact that it is more difficult to construct consistent patterns to de-identify locations. For example, while the algorithm checks for hospital name patterns using keywords such as "hospital", "medical center", and "clinic", many hospital names are in the form of acronyms (e.g., GH instead of General Hospital) and thus it is sometimes difficult to identify this type of (non-critical) PHI.

## Discussion

The de-identification software was developed to scrub patient and provider identifying information from MIMIC II free-text medical records before limited public release of the data under a data use agreement. This software has been used to de-identify approximately 700,000 nursing notes, 30,000 discharge summaries, and 300,000 radiology reports, containing a total of approximately 220 million words (~1.8 GB). Our algorithm, running on a standard desk-top computer (3.4 GHz Pentium 4 processor with 512 kb cache) can de-identify text at a rate of approximately 10 MB per hour. Large US hospitals produce terabytes of data annually, but typically only about 8 GB of this is free text data (22 MB/day) [30], so that real time de-identification of free text data from a major hospital seems quite feasible with our software. De-identification of large volumes of data can be even more rapidly performed using several multi-core servers, as is our current approach.

The algorithm recall performance on the gold standard corpus of re-identified nursing notes (with an average rate of 0.967) is better than the average individual human de-identifier (0.81), the best single human de-identifier (0.94), and the average consensus of two human de-identifiers (0.94). On the test data, the algorithm performs as well as the best single human de-identifier and the average consensus of two human de-identifiers.

The algorithm performed well with critical PHI, missing no patient names in each corpus. Only one instance of an age over 89 was missed in the gold standard data, and three in the test corpus. This indicates that number-related sub-routines performed well, evidenced by the fact that only two full dates were missed in the test corpus.

Locations proved more difficult to detect, with less than five and two false negatives per 100,000 words in the gold standard and test corpora respectively. It is interesting to note that when customized dictionaries are not used, the algorithm performs almost as well, except for locations, with more than 23 times as many incidences of PHI for this category. It is therefore important to make sure that an extensive local dictionary is used to ensure locations smaller than a state, and in particular hospital-specific names, are removed by the algorithm.

Word misspellings are a major source of difficulty in de-identifying free text. Extensive lists of known PHI are used to identify instances of names, locations, etc. No spell-checking libraries such as *Ispell *or *Aspell *[25] are used. However, misspelled PHI instances do not match these known PHI instances, and currently context information is used to identify them. For example, uncommon words preceded by "Mr." or "Doctor" are identified as name PHI. The false positive count rises when these context rules are too inclusive, while lax rules elevate the risk of PHI release. The rules for the algorithm were based on careful evaluation of the trade-off between the number of false negatives and false positives.

An obvious extension is to look for likely misspellings of the patient's name if known *a priori *as names are the most dangerous type of PHI to miss. Similarly, searches for single digit omissions, insertions and reversals in social security numbers, medical record numbers and other *a priori *known patient identifiers can be used.

In addition to the PHI categories specified by HIPAA, free-text context information may reveal a patient's identity. For example, "the patient's trailer was blown away by a tornado the night before Christmas" is a piece of text that does not contain any terms that are outright PHI, but the date is obvious to a human and a news search could potentially reveal details on this newsworthy event and the identity of this relatively unique patient. Despite the risk of such inadvertent PHI disclosure, it is not feasible to have a clinician review every de-identified record to ensure removal of all PHI. In fact, Table 2 illustrates that even a consensus of three expert de-identifiers was incapable of removing all PHI. A potential, but difficult avenue for further work would be to devise an intelligent method to scrub contextual information, such as in the above example, which can indirectly reveal a patient's identity.

Although machine learning approaches coupled with large and representative labelled databases could identify some PHI occurrences, such systems are innately fragile. The combination of machine learning techniques with sensible heuristics has been shown to improve the precision and recall of de-identification systems [28]. However, it is unlikely that all eventualities can be represented in either of these approaches. It is more likely that an information-identification approach will work more accurately and ultimately be of more use. That is, there is an assumption that every component of a medical text is intended to mean something to the person who wrote it. A classification problem that identifies the semantic categories of each component of the text can thus mark for exclusion as potential PHI (or irrelevant information) any element for which it has no contrary evidence.

Before such systems can be developed, gold standard corpora (encompassing a vast range of data types) must be created and made available. Until now no public database of free text elements of medical records has been available, and comparisons of the algorithm described here with other algorithms are therefore difficult. Brief testing of other publicly available algorithms produced poor results on the gold standard and test corpora because each algorithm is designed specifically for a particular type of data structure. The availability of de-identification algorithms, especially open-source algorithms that can be customized and extended, will make further development of large gold-standard corpora feasible.

Medical de-identification systems have the potential for widespread use in information sharing for research purposes. The system described here is sufficiently generalized to handle text files of almost any format, albeit with varying performance, and will be useful in other research groups' de-identification efforts and database construction. Each PHI identification module can be switched on or off and dictionaries can be changed or switched. Furthermore, the user is able to specify which word categories are to be identified as PHI. Thus, it is possible to identify the full range of PHI categories or any subset of it, making the software appropriate for use on any type of text or medical record. In the spirit of open-source software, the full source-code has been made available online for public use via PhysioNet [8,9].

It should be noted however, that de-identification does not supersede the Common Rule [31], which applies to any human subject research that draws from any confidential medical records and states the responsibility of researchers to obtain IRB approval prior to data collection, as well as the responsibilities of IRB's to ensure the data are used safely.

IRB's are allowed, in the case of "minimal risk" and significant benefit, to grant permission to use clinical data in research without explicit patient consent, if such consent would be impractical to get. For example, we received approval to use raw patient data to help develop de-identification algorithms, with various safeguards to make sure that the data do not "leak". Our algorithm, however, is not meant to subvert these rules, and as such, our algorithm is insufficient to remove all PHI types required by HIPAA, and human oversight is also probably required.

An alternative approach that virtually ensures full HIPAA-compliant de-identification is that of concept-matching [26] where the output is devoid of phrases that do not map to a reference terminology and is stripped of nonmedical and extraneous information. Although, some relevant information may be removed, concept matching also provides the terminology code for each medical term included in the sentence, making it possible to index and relate the terms to each other and standard biomedical ontologies.

## Conclusion

This article describes a de-identification algorithm that has a better recall rating than the average de-identification efforts of a consensus of two trained medical professionals, and significantly better recall than any one expert. In any de-identification system, there is a strong possibility that the software may encounter PHI that are absent in the extensive dictionaries of known PHI and that are also not identified by the rules. To reduce the risk of inadvertent PHI exposure, the MIMIC II (non-gold standard) de-identified text files are being released only to selected research groups who are required to sign data use agreements. As such, we do not recommend the open publication of after scrubbing with our algorithm. It should also be noted that without explicit IRB approval, even with a 'perfectly' accurate algorithm, publication of data may not be allowed. This is because potentially embarrassing or legally sensitive information (not explicitly prohibited under HIPAA) may be contained in text.

In the version of the software that has been released to the public, references to the doctor and patient names extracted from MIMIC II that are specific to the database have been excluded. Additionally, the re-identification module of the software has been excluded from the released version since this module provides a mapping between each PHI in the text file and the surrogate term with which it is replaced. All information essential to the algorithm's functioning has been included, however, including dictionaries of common words and certain Systematized Nomenclature of Medicine (SNOMED) terminology extracted from the UMLS, lists of first and last names, and other relevant sources.

The current release of the software is optimized for performance on MIMIC II nursing notes and discharge summaries from U.S. hospitals, and the gold standard corpus currently used to determine performance statistics includes only nursing notes. With textual medical records becoming increasingly important for medical research, de-identification and evaluation of different types of records is increasingly important. In future work it will be interesting to evaluate the current de-identification software on other medical records, e.g., X-ray, EKG and echo reports. By posting the source code (under an open source license) and the annotated data that was used to tune the algorithm [8], we hope that the algorithm will undergo further development and adaptation to a wide variety of free text records. Other groups are also invited to contribute to the software and databases to increase the variety of dictionaries and free text corpora available for de-identification evaluation.

## Abbreviations

*HIPAA*: Health Insurance Portability and Accountability Act; *MIMIC*: Multi-Parameter Intelligent Monitoring for Intensive Care; *PHI*: Protected Health Information; *SNOMED: *Systematized Nomenclature of Medicine; *UMLS: *Unified Medical Language System.

## Availability and requirements

The software and data for reproducing the results in this paper are available from PhysioNet at . Prospective users of the data are required to accept a data use agreement. The source code was implemented in Perl (version 5.8.8 and above) and tested under Fedora Core 6, Linux 2.6.18, and under Windows XP, but should be usable on any platform for which a current Perl interpreter is available. The source code has been released under the GNU Public License, version 2.

## Competing interests

The authors declare that they have no competing interests.

## Authors' contributions

IN contributed to the de-identification software by improving its precision and adding important supplementary filtering modules to process discharge summaries. IN also conducted performance evaluation of the software using the test data set. 

MMD co-designed and implemented the initial version of both the human and automated de-identification software. MMD was also the main contributor in the de-identification and re-identification of gold standard corpus.

LHL contributed to the de-identification software by improving its recall and precision performance and adding further filtering modules to process discharge summaries. LHL conducted studies related to the differential filtering performance evaluation, distribution of PHI categories in the gold standard corpus, and performance of the algorithm using partial dictionaries.

AR provided advice for MMD during development of the human expert de-identification review software. AR recruited, coordinated and trained the human expert de-identification clinicians. AR also performed expert adjudication for ambiguous results.

MV provided code support and testing of the algorithm on the MIMIC II database.

WJL provided natural language processing support and helped optimise the code for fast implementation on large databases.

PS provided advice and support for natural language and medical dictionary analysis.

GBM provided advice and support, particularly in the code review process, as well as input on algorithm design and evaluation.

RGM provided overall mentorship, clinical guidance and interpretation and review of ambiguities in clinical notes, as well as guidance on evaluation strategies.

GDC organized the project, provided mentorship for MMD, IN and LHL, reviewed and suggested code improvements, and helped design the test and evaluation strategy. GDC also provided background research and co-wrote the article with IN and LHL.

All authors reviewed and approved the article.

## Pre-publication history

The pre-publication history for this paper can be accessed here:



## Supplementary Material

Additional File 1 **Appendix**. A:  PHI Tag Types. B: Example Regular Expressions in Perl. C: List of Dictionary Files. Click here for file
