# Supplementary material for: Automated de-identification of free-text medical records
Source: BMC Med Inform Decis Mak. 2008 Jul 24;8:32. doi: 10.1186/1472-6947-8-32 (PMC2526997; doi:10.1186/1472-6947-8-32)
Supplement: Additional File 1 — Appendix. A: PHI Tag Types. B: Example Regular Expressions in Perl. C: List of Dictionary Files. [file 1472-6947-8-32-S1.doc]

# APPENDIX A. PHI Tag Types

The de-identification algorithm replaces each PHI found in the medical notes with a PHI category tag. In this section, we list the PHI tags defined in the code.

## Name

The name filter replaces each name instance found in the medical notes with a PHI tag that indicates the type of name replaced (e.g., first/last, female/male). In some cases, the pattern used to detect the name is specified in parenthesis following the name type. For example, the tag [*** Name (PTitle) ***] indicates that the name matches patterns defined by plural titles such as “Drs.” and “Professors”. Example name PHI tags are:

[** Known patient firstname **] Name matched the patient’s first name listed in the dictionary.

[** Known patient lastname **] Name matched the patient’s last name in the dictionary.

[** Doctor First Name **] Doctor first name.

[** Doctor Last Name **] Doctor last name.

[** Female First Name (un) **] Unambiguous female first name.

[** Male First Name (un) **] Unambiguous male first name.

[** Name (MD) **] Doctor names followed by “MD”.

[** Name (PRE) **] Doctor name preceded by words such as “physician”, “PCP”, “provider”, etc.

[** Name (NameIs) **] Name preceded by the term “name is”.

[** Name Prefix (Prefixes) **] Name prefixes such as “de la”, or “van der”.

[** Last Name (Prefixes) **] Name preceded by prefixes such as “de la” or “van der”.

[** Name (STitle) **] Name followed by specific titles, such as “DR”, “MR” or “MS”.

[** Name (PTitle) **] Name followed by plural titles such as “Drs.” And “Professors”.

## Location

PHI category tags generated by the location filters include the following.

[** Street Address **] Street address.

[** Location **] Location in general, such as town, city names.

[** Location (Universities) **] University names.

[** Hospital **] Hospital names.

[** Wardname **] Hospital ward names.

[** PO BOX **] PO Box number.

[** State/Zipcode **] Zipcode preceded by state names.

[** State **] U.S. state names.

[** Country **] Country name.

[** Company **] Company name.

## Telephone

The phone filter generates the following two types of PHI category tags.

[** Telephone/Fax **] Telephone or fax numbers.

[** Pager number **] Pager or beeper numbers.

## Miscellaneous

[** Social Security Number **] Social security numbers.

[** Medical Record Number **] Number associated with the medical record.

[** Unit Number **] Unique patient number.

[** Age over 90 **] Age equal to 90 or older.

[** E-mail address **] Email address.

[** URL **] Web URL address.

[** Holiday **] Holiday such as Christmas, Hanukah, Ramadan.

[** Ethnicity **] Words that indicate ethnicity or nationality, such as American, African, Spanish, etc.

## APPENDIX B. Example Regular Expressions in Perl

This appendix gives example regular expressions in the deid software in Perl syntax. Each expression is enclosed in a pair of “/” (i.e., */pattern/*). Expressions in square brackets represent a range of characters. The expression [0-9] indicates a digit. The expression “\d” matches numeric; numbers in a pair of curly braces following the expression indicate the number of digits for the match. For example, “\d{4}” matches a 4 digit number. The expression “\s” matches white space. The question mark indicates an optional expression; “+” matches the preceding pattern element one or more times; whereas “*” indicates a match for 0 or more times. The vertical bar “|” separates alternative expressions. The expression “\w” matches alphanumeric; “\b” matches word boundaries.

**Example 1**: The following regular expression checks for month/day/year date pattern, such as “03/06/2008” or “3-6-08”.

**/\b(\d\d?)[\-\/](\d\d?)[\-\/](\d\d|\d{4})\b/**

**Example 2**: The following regular expression checks for date patterns such as “3^rd^ of June” or “25^th^ December”, where $m contains a string that represents month of the year (such as "January", "Jan", "February", "Feb", etc.).

**/\b((\d{1,2})(|st|nd|rd|th|)?( of)?[ \-]\b$m)\b/**

**Example 3**: The following regular expression checks for PO Box number patterns, such as “P.O. Box 02139” or “PO BOX # 02139”.

**/\b(P\.?O\.?\s*Box\s*\#?\s*[0-9]+)\b/**

**Example 4**: The following regular expression checks for URL patterns that begin with the string “http” or “https”, such as “[http://www.mit.edu](http://www.mit.edu/)” or “[https://web.mit.edu](https://web.mit.edu/)”.

**/\bhttps?\:\/\/[\w\.]+\w{2,4}\b/**

**APPENDIX C. List of Dictionary Files**

This appendix describes dictionary files used by the de-identification software and the number of entries in each dictionary file.

## A Priori Surrogate Names and Locations

[pid_patientname.txt](http://www.physionet.org/physiotools/deid/lists/pid_patientname.txt)

163 full names and ids of the patients in the gold standard corpus

[doctor_first_names.txt](http://www.physionet.org/physiotools/deid/lists/doctor_first_names.txt)

56 given names of doctors

[doctor_last_names.txt](http://www.physionet.org/physiotools/deid/lists/doctor_last_names.txt)

254 family names of doctors

[stripped_hospitals.txt](http://www.physionet.org/physiotools/deid/lists/stripped_hospitals.txt)

143 names of nearby hospitals

[local_places_unambig.txt](http://www.physionet.org/physiotools/deid/lists/local_places_unambig.txt)

48 unambiguous names of nearby towns and cities

[local_places_ambig.txt](http://www.physionet.org/physiotools/deid/lists/local_places_ambig.txt)

4 ambiguous names of nearby towns and cities

## Generic Names

[last_names_unambig.txt](http://www.physionet.org/physiotools/deid/lists/last_names_unambig.txt)

81,497 unambiguous family names

[last_names_ambig.txt](http://www.physionet.org/physiotools/deid/lists/last_names_ambig.txt)

7,298 ambiguous family names

[last_names_popular.txt](http://www.physionet.org/physiotools/deid/lists/last_names_popular.txt)

93 popular family names

[prefixes_unambig.txt](http://www.physionet.org/physiotools/deid/lists/prefixes_unambig.txt)

17 family name prefixes (von, de la, etc.)

[last_name_prefixes.txt](http://www.physionet.org/physiotools/deid/lists/last_name_prefixes.txt)

138 prefixes that may appear before a family name

[female_names_unambig.txt](http://www.physionet.org/physiotools/deid/lists/female_names_unambig.txt)

3843 unambiguous female given names

[female_names_ambig.txt](http://www.physionet.org/physiotools/deid/lists/female_names_ambig.txt)

616 ambiguous female given names

[female_names_popular.txt](http://www.physionet.org/physiotools/deid/lists/female_names_popular.txt)

125 popular female given names

[male_names_unambig.txt](http://www.physionet.org/physiotools/deid/lists/male_names_unambig.txt)

1144 unambiguous male given names

[male_names_ambig.txt](http://www.physionet.org/physiotools/deid/lists/male_names_ambig.txt)

419 ambiguous male given names

[male_names_popular.txt](http://www.physionet.org/physiotools/deid/lists/male_names_popular.txt)

130 popular male given names

## Generic Locations

[countries_unambig.txt](http://www.physionet.org/physiotools/deid/lists/countries_unambig.txt)

179 country names

[us_states.txt](http://www.physionet.org/physiotools/deid/lists/us_states.txt)

59 US states and territories

[us_states_abbre.txt](http://www.physionet.org/physiotools/deid/lists/us_states_abbre.txt)

59 standard US state and territorial abbreviations

[more_us_state_abbreviations.txt](http://www.physionet.org/physiotools/deid/lists/more_us_state_abbreviations.txt)

53 non-standard US state name abbreviations

[locations_unambig.txt](http://www.physionet.org/physiotools/deid/lists/locations_unambig.txt)

3341 unambiguous location names

[locations_ambig.txt](http://www.physionet.org/physiotools/deid/lists/locations_ambig.txt)

135 words that may be (parts of) location names

## Other possible PHI

[us_area_code.txt](http://www.physionet.org/physiotools/deid/lists/us_area_code.txt)

382 US telephone area codes

[company_names_unambig.txt](http://www.physionet.org/physiotools/deid/lists/company_names_unambig.txt)

484 unambiguous company names

[company_names_ambig.txt](http://www.physionet.org/physiotools/deid/lists/company_names_ambig.txt)

18 ambiguous company names

[ethnicities_unambig.txt](http://www.physionet.org/physiotools/deid/lists/ethnicities_unambig.txt)

195 ethnicities

## Dictionaries of Common Words and Medical Terms

This section describes dictionaries that contain lists of words and phrases that are not likely to be PHI

[common_words.txt](http://www.physionet.org/physiotools/deid/dict/common_words.txt)

49,668 words that are common in medical records

[commonest_words.txt](http://www.physionet.org/physiotools/deid/dict/commonest_words.txt)

5,126 words that are very common in medical records

[medical_phrases.txt](http://www.physionet.org/physiotools/deid/dict/medical_phrases.txt)

28 medical phrases

[notes_common.txt](http://www.physionet.org/physiotools/deid/dict/notes_common.txt)

66 very common words found in nursing notes

[sno_edited.txt](http://www.physionet.org/physiotools/deid/dict/sno_edited.txt)

175,313 medical terms from UMLS/SNOMED
